# Response of compacted bentonite to hyperalkalinity and thermal history

**DOI:** 10.1038/s41598-021-95023-5

**Published:** 2021-07-29

**Authors:** Rohini C. Kale, Bhanwariwal Kapil, K. Ravi

**Affiliations:** Department of Civil Engineering, Indian Institute of Technology, Guwahati, 781039 India

**Keywords:** Engineering, Civil engineering

## Abstract

The use of compacted bentonite around the high-level nuclear waste canister (HLW) inside the deep geological repository (DGR) ensures the prevention of entry of active radionuclides in the atmosphere due to its noteworthy large swelling ability. In the eventual repository, the waste canister has a high (100 °C–200 °C) temperature initially, and it reduces over a vast period, which induces a thermal history over the compacted bentonite layer. The cement/concrete layer is constructed as a bulkhead or in the vaults or to support the access of galleries between a buffer and the host rock, and it degrades over the period. The hyperalkaline fluid is created when it percolates through the cement/concrete layer and comes in contact with the compacted bentonite. The contact of hyperalkaline fluid to compacted bentonite induced with thermal history can hamper the swell pressure characteristic of the bentonite. Therefore to determine the combined effect of hyperalkalinity to the thermal history induced compacted bentonite, swell pressure testing has been conducted on two compacted Barmer bentonites (B_1_ and B_2_) specimens with an initial dry density of 1.5 Mg/m^3^, 1.75 Mg/m^3^, and 2.0 Mg/m^3^ and saturated with distilled water as well as with hyperalkaline cement water (W/C = 1 und pH = 12.5) and heated to 110 °C and 200 °C. When the specimens were saturated with hyperalkaline cement water, the swell pressure exerted by both bentonites was noticeably reduced compared to specimens saturated with distilled water. Nevertheless, the time taken to full saturation was longer than distilled water for samples saturated with hyperalkaline cement water. Also, the decrease in swell pressure was observed in the samples subjected to thermal history than samples, which were tested without inducing thermal history in both the cases of hyperalkaline cement water and distilled water. The microstructural observations through XRD, FESEM and EDX revealed the clogging of pores due to the presence of non-swelling minerals.

## Introduction

A Deep Geological Repository (DGR) consisting of a multi-barrier structure is the most attentive option for high-level radioactive waste (HLW) disposal^1–3^. The multi-barrier structure comprises of an existing host rock and buffer material for the waste canister. The buffer material is required to perform the isolation of the waste canister from the environment. The stable performance of the whole system is expected under the significant atmospheric changes and hydrophobic forces; therefore, it should be positioned at a suitable geological depth^4,5^. Bentonite is a suitable buffer material that is used to seal the waste canister and is compacted between the existing rock and the waste canister due to its huge swelling potential and low permeability^6,7^. The protection of the DGR depends on geological stability and the stability of bentonite buffer. According to^8,9^, in Callovo-Oxfordian (COX) clay formations, the DGR in the French concept will be installed, and rock buffer interface safe temperature should be less than 90 °C. Comparatively, in the Swiss concept, the DGR will be constructed in Opalinus (OPA) argillaceous formations, and the safe temperature of the rock buffer interface should be 75 °C–95 °C^10^. According to^11^, the expected temperature of the canister surface was 160 °C, and it decreased to temperatures below 100 °C within around 200 years. The bentonite buffer, which is placed at the interface between the geological formation and the waste canister, controls the thermal load from both sides^12^. As the waste canister emits a very high temperature while placing it in the repository, the surrounding layer of bentonite experiences heat over the operational period of the repository. In reported literature, the impact of temperature on compacted bentonites has been understood by several experiments. According to^13^, the temperature of the waste canister would increase the chances of alterations in compacted bentonite buffer during its unsaturated phase^14^ had modeled the physicochemical characteristics of bentonite in the context of deep geological repository under 200 °C and 100 °C temperatures. The results of simulations showed the enhanced amount of illitization in the bentonite buffer as the temperature increased. The hot canister surrounded by the buffer experiences a continuous heating effect until the radioactivity of the element reduces over thousands of years. For the safety evaluation of the repository design, the maximum temperature of the canister was considered 150–200 °C^15^. The temperature decreases with time, i.e., with reduction of the radioactive activity, and thus the temperature drop induces a thermal history on the compacted bentonite over a long time. Very few researches have reported on the role of thermal history and its effect on the behavior of bentonite^16,17^.

A layer of cement or a concrete bulkhead^18,19^ is a part of the multi-barrier system that may be used either as a massive embedding^20,21^ or in the vaults as a backfill^22^ or as mechanical support to a repository and backfilling of cavities^23,24^ or to support the access galleries and sealing of the final path^21^. The continuous degradation of cement of the concrete layer produces a highly alkaline environment (pH > 12) inside its compact structure. This cement layer comes in contact with the moisture that percolates from the host rock and forms hyperlkaline solutions that will try to diffuse through the compacted bentonite buffer. Due to the hyperalkaline environment, the properties of bentonite such as swell pressure, permeability can be affected, and the reactive transportation process can be regulated via the compacted bentonite^25,26^. It can also improve the solubility of radioactive elements by impeding their swelling and permeability^27^. Several reports on the effects of the alkaline atmosphere on the properties of compacted bentonite have been published in the past^28–30^. The interaction of hyperalkalinity with expansive clay was experimentally studied by^18^. There was a minor reduction in hydraulic conductivity and a marginal change in the dissolution of minerals due to the interactions of cement water. Cuisinier et al.^31^ studied the influence of the hyperalkaline environment on the properties of bentonite buffer material by saturating samples with cement water for various periods. It was observed that the circulation of cement water increased the macropore void ratio 1.5 times for the MX-80 bentonite. Jenni et al.^32^ conducted a series of studies to explore the interaction of ordinary Portland cement and Opalinus clay and reported that the porosity at the cement clay interface changed, and the pores were clogged by reactive transport of diffusive solute fluxes and various reaction kinetics.

Not only the experimental work but also a modeling of cement clay interaction was reported in previous studies by some of the researchers^33–35^. Watson et al.^24^ reported a reactive transport model of a large-scale, in situ, long-term cement/clay system. The ion exchange and the dissolution of portlandite, calcite, and ettringite in concrete were stated to play a significant role. The deposition and degradation of clay minerals were also observed on the concrete bentonite interface. The reaction transport from the bentonite to the cement pores was modeled using PRECIP by^33^. Minerals were allowed to dissolve and precipitate with the help of the reaction mechanism. Analyses were carried out using various cement pore fluids compositions, rates of growth of minerals, dissolutions of minerals such as montmorillonite, the solubility of minerals, etc. It was studied that the transport of pore moisture through the compacted bentonite was the main reason for the alterations that was occurred. The bentonite possesses swelling potential due to the presence of a mineral called montmorillonite^36,37^. It is an essential characteristic of bentonite as it fixes the gaps between the layers of bentonite and the fissures between the host rock and bentonite layer and suppresses the microbial activity^38^. Several reports have been published regarding the bentonite swelling under the influence of strongly alkaline solutions^39,40^. He et al.^41^ found that when the compacted bentonite had come in contact with highly alkaline fluids, the swell pressure was reduced. Conclusion given by^42^, which mostly resulted from the dissolution of montmorillonite and precipitation of secondary minerals, registered a decrease in swell pressure of GMZ bentonite when reacted with a highly alkaline solution.

The protection of the high-level nuclear waste repository is based on a multi-barrier structure, and the bentonite buffer plays a crucial role in the designed barrier system. One key aspect that can fulfill the function of bentonite as a buffer is its swelling capacity. Though the past reported studies had helped to understand the behavioral change in bentonite under high temperature conditions or under alkaline environment, the combined effect hyperalkaline environment under thermal history was the main focus of this study, which a bentonite layer has to undergo in the eventual repository and not reported in the past. Hence, this article analyzed the swelling pressure of compacted bentonite, saturated with hyperalkaline cement water, induced with different thermal histories. The results were compared with the specimens induced with thermal history and saturated by distilled water^16^ that should have a tangible contribution to the study of nuclear waste disposal.

## Materials

This study employed two bentonites (B_1_ and B_2_) from the Barmer district of Rajasthan, India. Procedures explained by^17^ were used to determine the initial characterization of both bentonites (Table 1). Bentonite powders were microstructurally examined using the electron scanning microscope (FESEM), X-Ray Diffraction (XRD) and the Energy Dispersive X-ray test (EDX).

**Table 1:** Properties of bentonites used in the study^17^.

Properties	Barmer 1	Barmer 2
Specific gravity	2.79	2.77
Clay content (%)	89	76
Sand sized fraction (4.75–0.075 mm)	1.63	3.70
Liquid limit (%)	447.28	248.92
Plastic limit (%)	49.23	40.90
Plasticity Index (%)	398.05	208.02
Specific surface area (m^2^/g)	507.74	273.9
Maximum dry density (g/cm^3^)	1.42	1.18
Optimum moisture content (%)	35	33
Cation exchange capacity (meq/100 g)	94.58	64.07
Free swell index (%)	833.33	693.33
pH	8.29	8.47

### Methodology

#### Swell pressure

**Figure 1:**
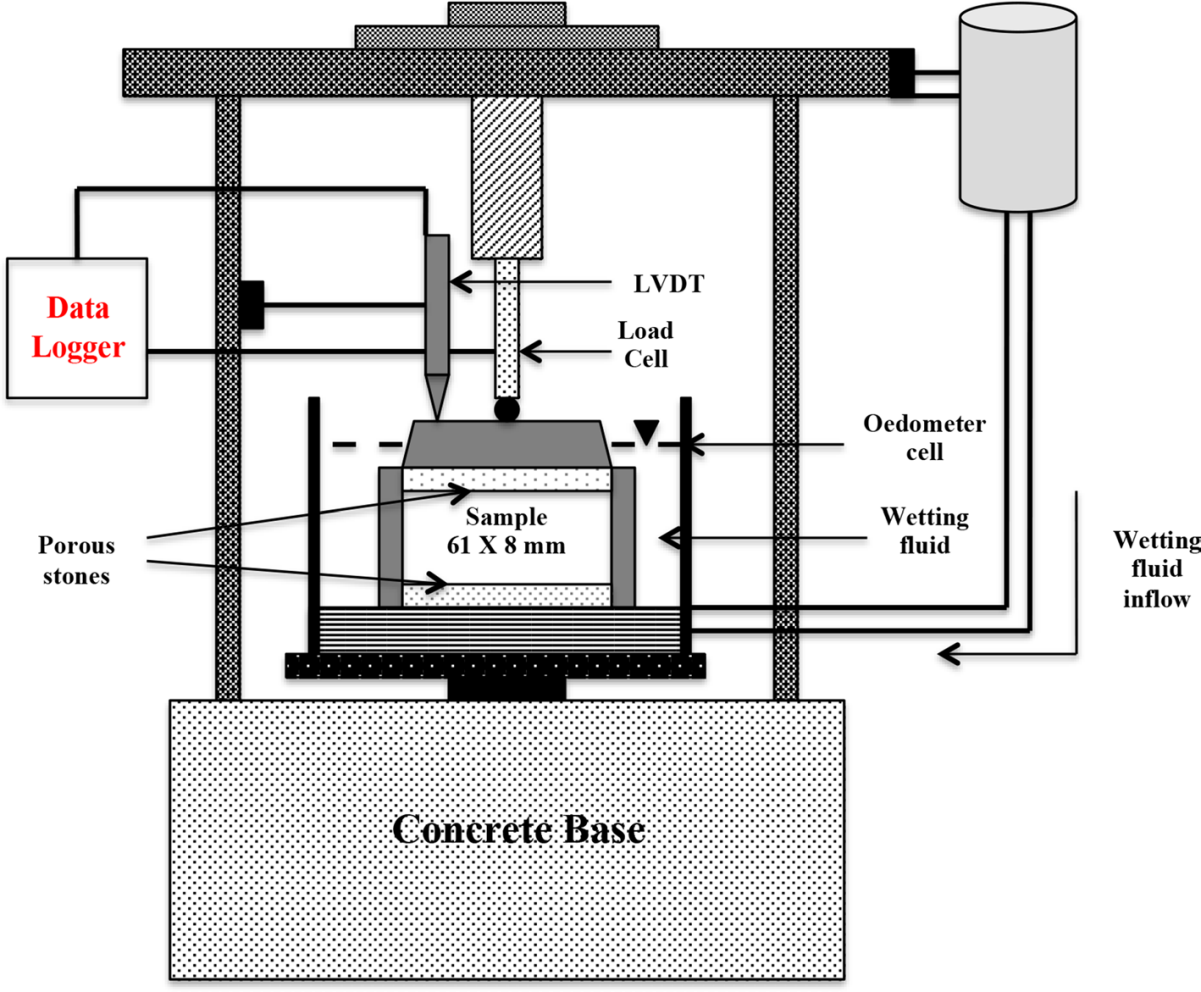
Schematic representation of swell pressure set up (Kale and Ravi, 2019).

The oedometer was used to calculate the swelling pressures of compacted bentonite specimens^43^. Air-dried samples of B_1_ and B_2_ were compacted in triplicates in an oedometer ring with diameter = 61 mm, and height = 8 mm. The initial water content of B_1_ and B_2_ were 14% and 11%, respectively, and three separate compaction densities, i.e., 1.5 Mg/m^3^, 1.75 Mg/m^3^, and 2.0 Mg/m^3^, were taken into consideration for the evaluation. The swell pressure assembly was a 10 kN loading cell with 0.01 kN sensitivity and the Linear Variable Differential Transformer (LVDT) with a maximum 20 mm displacement and a sensitivity of 0.01 mm, which was mounted over the oedometer ring. The specimens were saturated using cement water. A schematic representation of the test setup is shown in Fig. 1. The load cell recorded various load values during successive time periods of saturation as soon as the test began and the sample came into contact with the moisture. The procedure was stopped after a steady reading was seen in the load cell, indicating saturation^44^. The LVDT estimated displacement was virtually nil over the whole test period, as the swelling was in a constant volume state. The load divided by the area of cross-section of the specimen at each interval gave the swell pressure value of the compacted bentonite specimen at each time interval as well as the final value after the saturation.

#### Preparation of cement water (CW)

The ordinary Portland cement powder was mixed in an equal ratio with distilled water (1:1)^18^. The powder was properly mixed and permitted to settle down to extract and filter the supernatant water. The cement water (CW) used in this experimental study had a pH value of 12.5 that was hyperalkaline. Table 2 lists the chemical composition of the cement solution.

**Table 2:** Chemical composition of cement water (CW) (mg/L).

Si	Al	Mg	Na	Ca	K	Fe	Cl	SO_4_	Cu	F	NO_3_	pH
218.337	44.64	0.1625	1320.453	658.285	921.225	0.2365	3.3234	1910.34	2.5318	1.4293	0.7035	12.5

#### Thermal history on the samples

The effect of cement water (CW) on swell pressure of all thermal history induced bentonite specimens (B_1_ and B_2_), compacted at various densities (1.5 Mg/m^3^, 1.75 Mg/m^3^, 2 Mg/m^3^) and hydrated by cement water (CW) have been studied in a series of experiments. The compacted bentonite specimens were exposed to a temperature of 110 °C and 200 °C for 3 h. The samples were removed from the oven after inducing the thermal history and used for testing. After the procedure, the findings were compared of unheated specimens of the same density.

## Results and Discussion

### Effect of hyperalkaline environment and thermal history on swell pressure

#### Time swelling of compacted bentonites saturated with cement water

**Figure 2:**
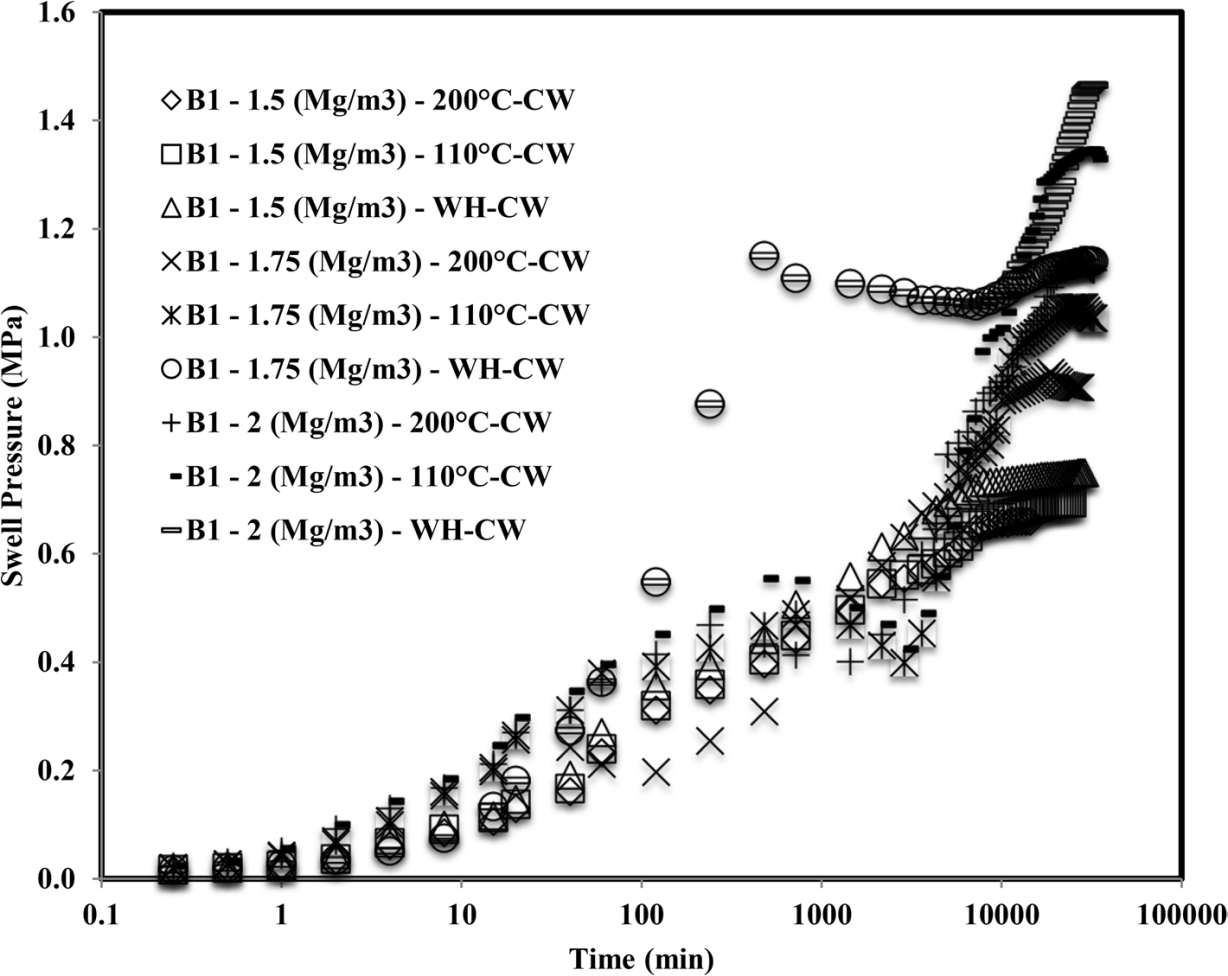
Time swelling curve of B_1_ bentonite when heated at different temperatures saturated with cement water (CW).

**Figure 3:**
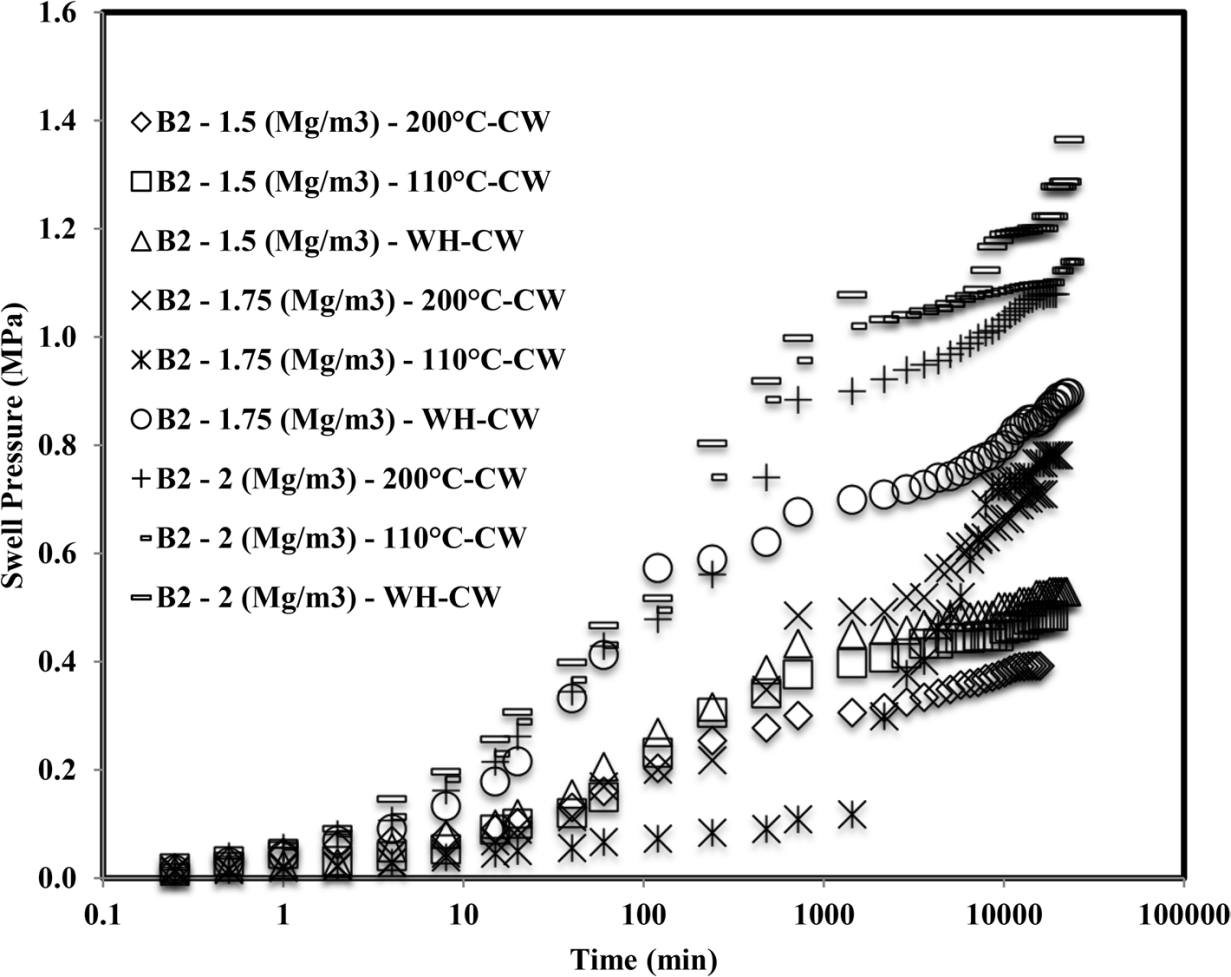
Time swelling curve of B_2_ bentonite when heated at different temperatures saturated with cement water (CW).

Figure 2 shows the plot of swell pressure vs. time for bentonite B_1_ induced with thermal history and saturated with the cement water (CW). The saturation time of the sample compacted at 1.5 Mg/m^3^ was 28,800 min, 24,480 min, and 15,120 min, respectively, for 110 °C and 200 °C, without inducing thermal history. Similarly, sample compacted at 1.75 Mg/m^3^, without inducing thermal history, 110 °C and 200 °C, the saturation time was observed as 36,720 min, 33,840 min, and 27,360 min, respectively. In addition, the saturation period of samples compacted at 2 Mg/m^3^ was observed as 43,920 min, 39,600 min, and 38,160 min respectively for thermal history free, 110° and 200 °C. Figure 3 depicts the plot of swell pressure vs. time of bentonite B_2_ for all three compaction densities and the thermal histories. For the sample compacted at 1.5 Mg/m^3^, it had been found that the period was 22,320 min, 18,720 min, and 15,840 min, respectively, with no thermal history, 110 °C and 200 °C. The time of saturation for samples compacted at 1.75 Mg/m^3^ had been noted as 24,480 min, 20,160 min, and 16,560 min, respectively, without inducing thermal history, 110 °C or 200 °C. The sample was also compacted to 2 Mg/m^3^, and the saturation times were 31,680 min, 24,480 min, and 19,440 min, respectively, without inducing thermal history, 110 °C or 200 °C.

The formation of the double peak was observed when both the bentonites were subjected to saturation with cement water (CW). The first peak might be observed due to the density at which the samples were compacted and the elastic network that was built up by the bentonite particles at the particular density. Initially, when the bentonite came in contact with the cement water (CW), the swelling occurred due to crystalline swelling^45^. The elastic nature of compacted bentonite would still have a strong structural bond due to interlamellar bonding. Hence it started swelling as well as creating pressures on the adjacent clay units. After the first peak, it got a relaxed for the deformation and the displacement, and hence for some time after the first peak, the swell pressure reduced slightly, or in some cases, it was constant. With the further ingression of cement water (CW), the clay particles tried to form a homogeneous structure by rearranging themselves and therefore creating an unbalanced net charge. In order to balance these unbalanced net charges, the bentonite started swelling and tried to reach saturation by moisture intake^46^. Therefore this second phase after rearrangement of particles gave rise to a secondary peak.

Smectite is a silicate with a 2:1 layer where the two tetrahedral silica sheets are bound to all sides of a gibbsite-like sheet of aluminum octahedral. The stability of smectite is distracted when pH > 12^47^. Strong hydroxyl anion concentrations (OH-) permit monomers and dimmers to form in solution with the cations present on the smectite layer. The high pH dissolves the silica from the smectite layer, precipitating the hydroxy-aluminosilicates and other minerals^48^. The diffuse double layer thickness reduction influenced the interparticle spread^49^. With a penetration of a highly alkaline solution, fringe-like colloids might grow on the lamellar surface of the montmorillonite, which could block the pores under constant volume conditions, reducing their hydraulic conductivity. Montmorillonite could be dissolved and fragmented in a highly alkaline solution; the expandable mineral might become less or non-expandable, which thus reduced its swell pressure^21^. Chen et al.^50^ detected the hydrothermal expansion and mineral change in the GMZ bentonite microstructure. The reduction in the swell pressure was one of the observations, which resulted due to the pore clogging by precipitation of calcium silicate hydrate gels on the surface of montmorillonite units.

#### Swell pressure of compacted bentonites subjected to thermal history

The swell pressure vs. temperature (of inducing thermal history) variation of the bentonite B_1_ and B_2_ saturated with cement water (CW) is presented in Table 3 for three different densities 1.5 Mg/m^3^, 1.75 Mg/m^3^, 2 Mg/m^3^, and the water contents of the specimens after applying the thermal history are presented in Table 4. The reduction in the final swell pressure values was observed with the increase in the temperature for all compacted densities and both the bentonites. It was observed that the decrease in the swell pressure value was higher at lower compaction densities than higher one (2 Mg/m^3^).

**Table 3:** Final swell pressure values of both the bentonites at each temperature for various densities.

Bentonite	Temperature (°C)	Swell pressure (MPa) at dry densities 1.5, 1.75 and 2.0 Mg/m^3^		
		1.5 (Mg/m^3^)	1.75 (Mg/m^3^)	2 (Mg/m^3^)
B_1_	200	0.661	0.948	1.143
	110	0.694	1.055	1.345
	25	0.75	1.14	1.472
B_2_	200	0.392	0.485	0.53
	110	0.71	0.781	0.896
	25	1.08	1.139	1.485

**Table 4:** The water contents of the specimens after subjected to thermal history.

Sr no	Bentonite	Density (Mg/m^3^)	Water content at 200 °C after 3 h	Water content at 110 °C after 3 h
1	B_1_	1.5	7.02	7.45
		1.75	7.27	8.89
		2	8.12	10.04
2	B_2_	1.5	8.08	8.96
		1.75	8.56	10.12
		2	8.98	11.04

At high temperatures, i.e., 200 °C, the crystalline swelling gets weak due to the adsorption of the water layer (Table 4). The function of thermal osmosis was subjected to that of the contraction lattice of the compacted bentonite after inducing thermal history, due to the limited DDL growth, at higher compaction density and constant volume conditions^51^. As a result, the final swell pressure at 200 °C in the present study is lower than 110 °C than specimens without inducing thermal history. However, past studies had pointed out the lower percentage of reduction in the swell pressure values when the confining stress, i.e., compaction density, was very high^52^. The macropores moisture at high density was transferred to microspores moisture due to an increase in the temperature that occupied the higher volume. Hence the smaller decrease in the swell pressure value was observed at higher density than the lower ones.

### Comparison of results of the specimens saturated with cement water and distilled water

#### Comparison of swell pressure for the specimens saturated with cement water and distilled water

**Figure 4:**
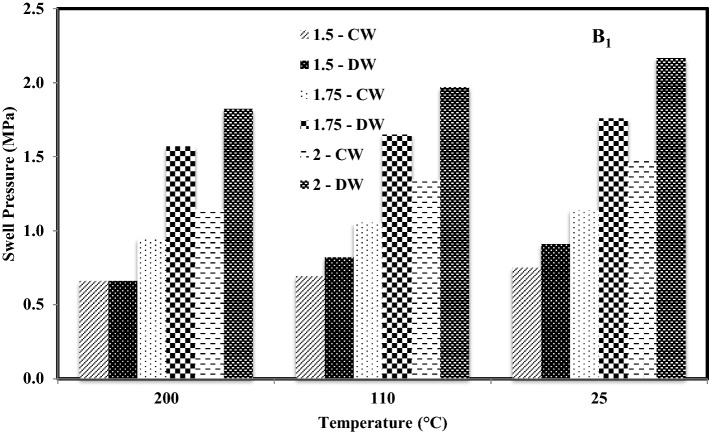
Comparison of variation in swell pressures of B_1_ with temperature and density saturated with distilled water^16^ and cement.

**Figure 5:**
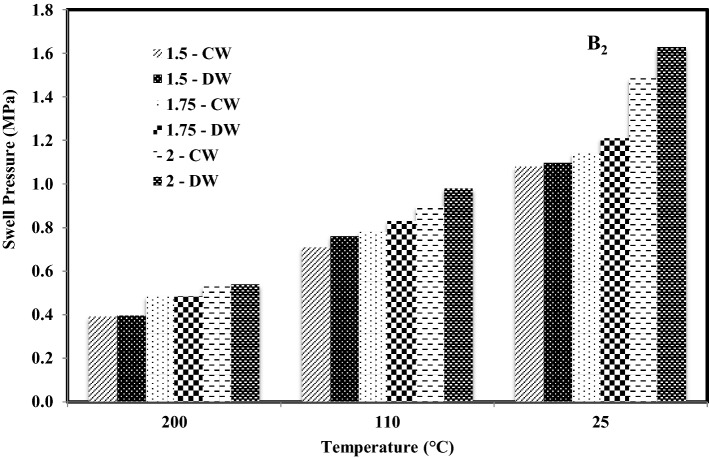
Comparison of variation in swell pressures of B_2_ with temperature and density saturated with distilled water^16^ and cement.

Figures 4 and 5 represent a comparison of variation in swell pressures of both bentonites with temperature and density saturated with cement water (CW) and distilled water (Kale and Ravi, 2019), respectively. It was observed that the swell pressure of bentonite compacted at 1.5 Mg/m^3^ and saturated with cement water and distilled water was 0.661 MPa and 0.662 MPa respectively when heated at 200 °C. Whereas it was 0.694 MPa and 0.821 MPa, respectively, when heated at 110 °C and 0.750 MPa and 0.909 MPa respectively for samples without inducing thermal history.

However, the swell pressure of bentonite compacted at 1.75 Mg/m^3^ and saturated with cement water (CW) and distilled water was 0.907 MPa and 1.570 MPa, respectively, when heated at 200 °C. Whereas it was 1.035 MPa and 1.650 MPa respectively when heated at 110 °C and 1.123 MPa and 1.760 MPa respectively for samples without thermal history and the swell pressure of bentonite compacted at 2 Mg/m^3^ and saturated with cement water and distilled water was 1.122 MPa and 1.823 MPa respectively when heated at 200 °C. It was reported as 1.320 MPa and 1.969 MPa, respectively, when heated at 110 °C and 1.448 MPa and 2.166 MPa, respectively, for samples without inducing thermal history. A similar trend was noted in the case of bentonite B_2_, and it was observed that the swell pressure in the case of bentonite B_1_ was recorded to be more than that of B_2_ when saturated with both the solutions. For both bentonites, the swell pressure in the case of cement water was less than the swell pressure in the case of distilled water.

In this particular reaction of cement water (CW) with the montmorillonite mineral, two important reactions were taking place. 1) The hyperalkaline solution attacked the structure lattice pattern of the montmorillonite mineral^53^. 2) On the further reaction of Ca(OH)_2_ with water, it formed free Ca^2+^ ions with OH^-^ ions, and these Ca^2+^ ions might have occupied the space between the interlamellar gaps of bentonite by the exchange of cations.

1 $${\text{Ca}}\;\left( {{\text{OH}}} \right)_{{2}} {\text{ + H}}_{{2}} {\text{O}} \Rightarrow {\text{Ca}}^{{{2} + }} + {\text{2OH}}^{ - }$$

These exchanges of cations might have reduced the interlamellar spacing of montmorillonite and therefore reducing the swell pressure of the bentonite. However, the OH^-^ ions being an anion migrated far away from the smectite lattice. As there was less possibility of an anion entering the narrow interlamellar space of the smectite and reacting with the cation from the specific surface, the probability of complete breaking down the structure lattice was low^53^. But the cement water (CW) being an OH^-^ dominant (pH > 12), could easily perform the dissolution of montmorillonite, resulting in lower swell pressure values^40^.

#### Comparison of final time of saturation in case of cement water and distilled water for different thermal history

**Figure 6:**
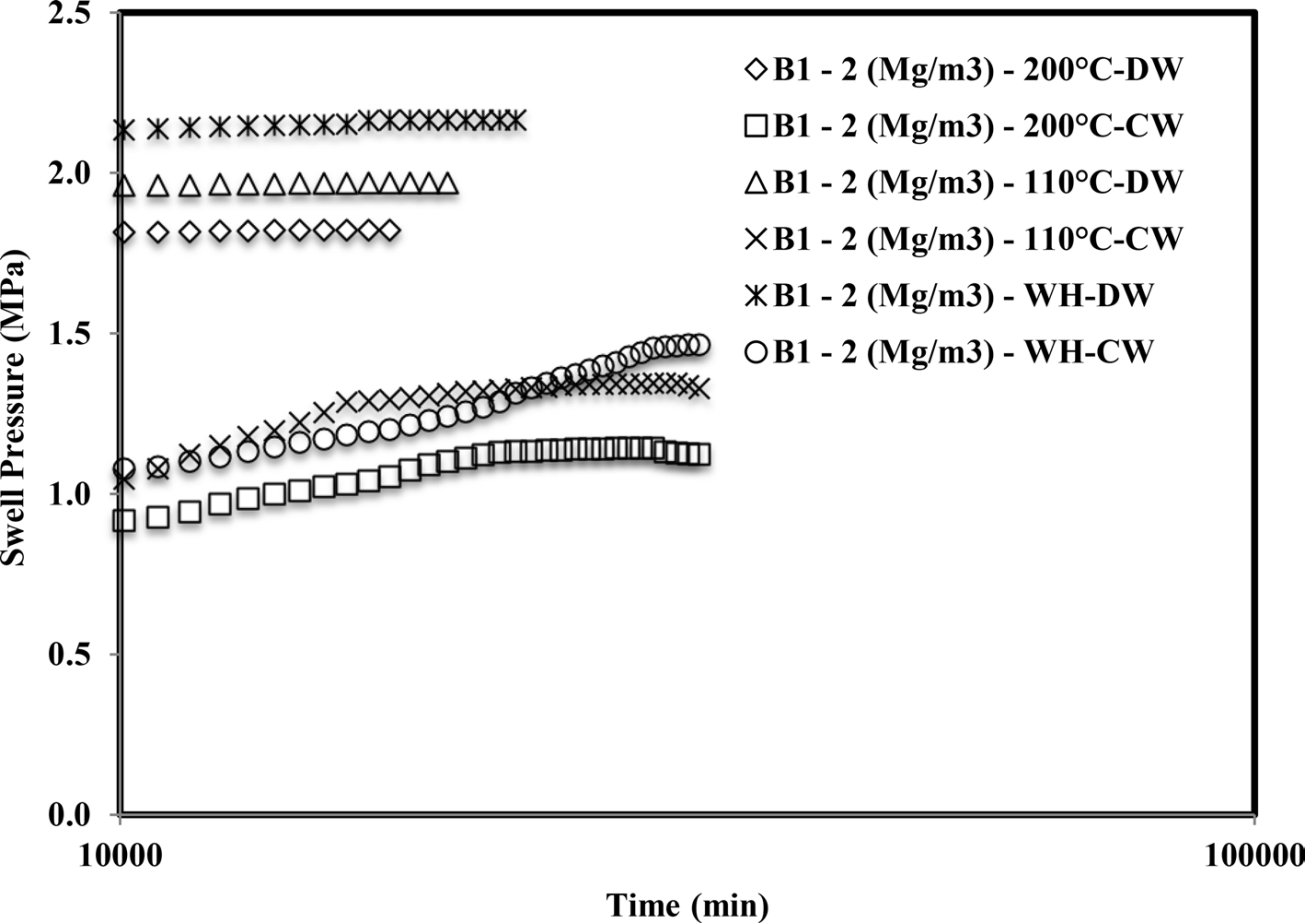
Comparison of variation of time of saturation of compacted bentonite B_1_ saturated with distilled water^16^ and cement water.

**Figure 7:**
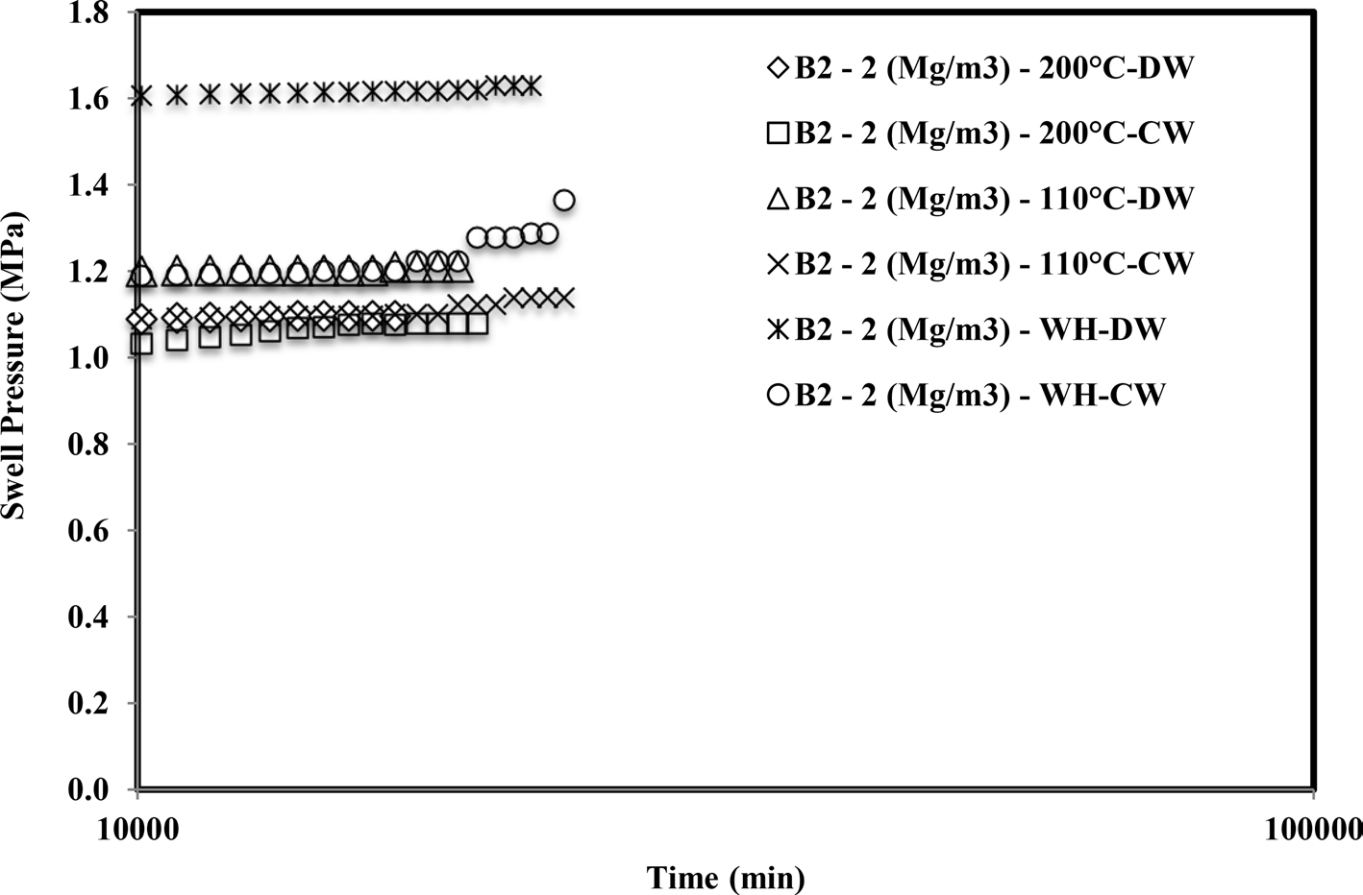
Comparison of variation of time of saturation of compacted bentonite B_2_ saturated with distilled water^16^ and cement water.

There is a comment on the time of saturation of compacted bentonites when saturated with cement water (CW) and distilled water (DW) (Figs. 6 and 7). Although the swell pressure of both bentonites was higher when saturated with distilled water than cement water, the time required for the saturation was longer in the case of cement water for all densities and thermal histories. The various samples were observed under a field emission scanning electron microscope (FESEM), and the presence of some non-swelling minerals in the pores of bentonites was observed. It is also supported with the help of XRD curves.

#### Comparison of initial time of saturation in case of cement water for different thermal history

**Figure 8:**
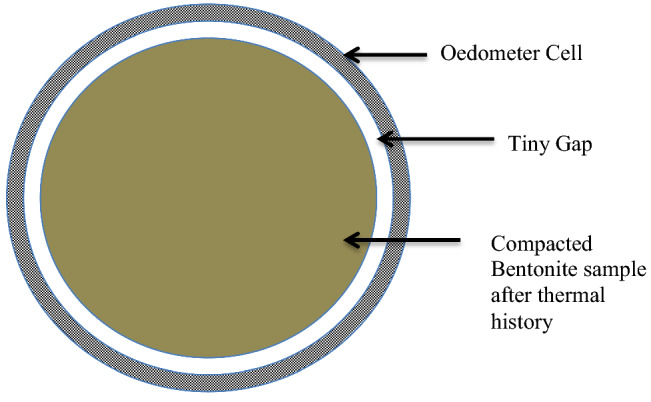
Representation of a tiny gap.

There was an observation regarding initial time of saturation of the samples. The initial time of saturation of all samples heated at 200 °C, 110 °C, and WH was compared, and it was observed that for the first 10 min of swelling of the sample, which was without inducing thermal history showed, the faster swelling than the sample heated at 110 °C than 200 °C. The compact specimens shrank, and a gap was found between the oedometer and the bentonite specimens as they were exposed to different thermal histories (Fig. 8). This gap was due to the evaporation of the water while inducing the thermal history, which could be considered as the pre-consolidation of the specimen. According to^54–56^, the cyclic wetting and drying of expansive clays might induce permanent plastic strains in the pore-network of the compacted specimens. Comparatively, the thermal histories in the specimens of the current study might have experienced this plastic strain in the microstructure of compacted bentonite. As soon as it came in contact with the saturating solution, the tiny gap might have filled first as the bentonite got the free space to expand. Once it touched the boundary of the oedometer ring, it exerted the vertical pressure on the load cell. Therefore, the swell pressure didn’t raised suddenly for the first 10 min for samples heated at 110 °C than 200 °C than the samples without inducing thermal history.

**Figure 9:**
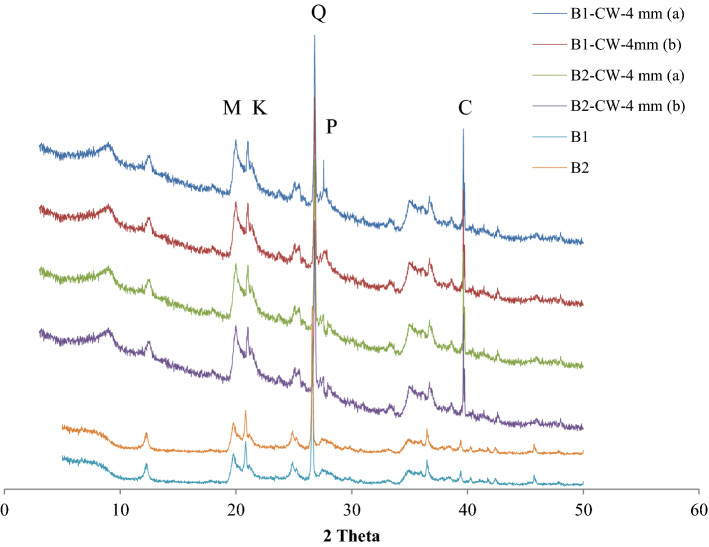
XRD analysis of bentonites. *M* montmorillonite, *K* kaolinite, *Q* quartz, *P* portlandite, C calcite.

Figure 9 depicts the XRD pattern of both bentonites saturated with cement water and distilled water. The 8 mm sample was cut into two parts, the lower part was indicated by 4 mm (a), and the upper part was indicated by 4 mm (b). The lower section of the sample in contact with the cement water showed a peak of portlandite mineral, while it wasn’t observed in the upper portion of the sample and bentonite B_2_. Samples saturated with cement water rather than distilled water samples were found to have the peak of calcite.

**Figure 10:**
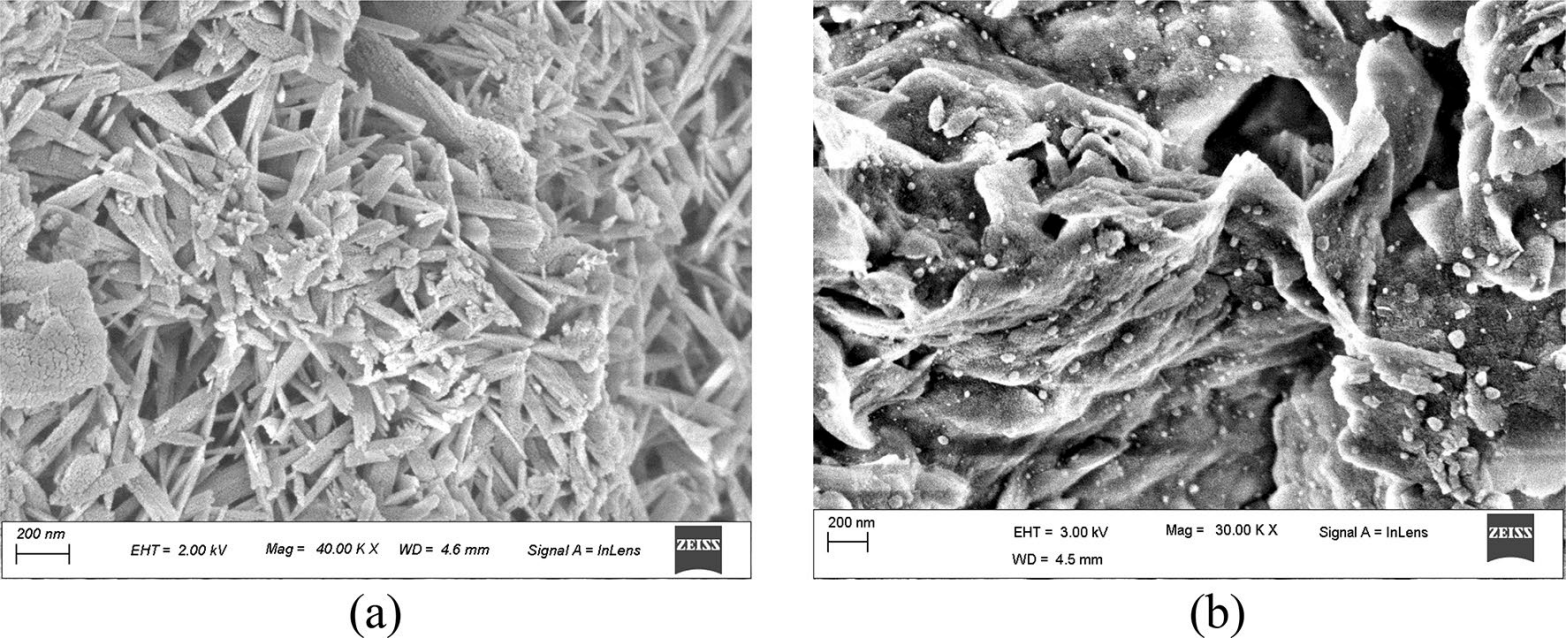
(**a**) Traces of Portlandite between the bentonite pores. (**b**) Calcite deposition on the flakes of bentonite.

Figure 10a,b is a FESEM image of a bentonite sample compacted at 2 Mg/m^3^ and saturated with cement water. It was observed that the plate-shaped structure was deposited between the two lamellar of bentonite, and it was probably a non-swelling mineral called portlandite. The portlandite mineral was formed when calcium from cement reacted with water.

2 $${\text{Ca}} + {\text{2H}}_{{2}} {\text{O}} \to {\text{Ca}}\;\left( {{\text{OH}}} \right)_{{2}} \;\left( {{\text{aq}}} \right) + {\text{H}}_{{2}} \left({\text{g}} \right)$$

The alkaline nature of the pore water that was present in the concrete was due to the presence of portlandite (pH = 12.5). The precipitation of calcite was also observed in FESEM images of some bentonite samples (B_2_). From Fig. 10b, the calcite deposition could be clearly observed.

**Figure 11:**
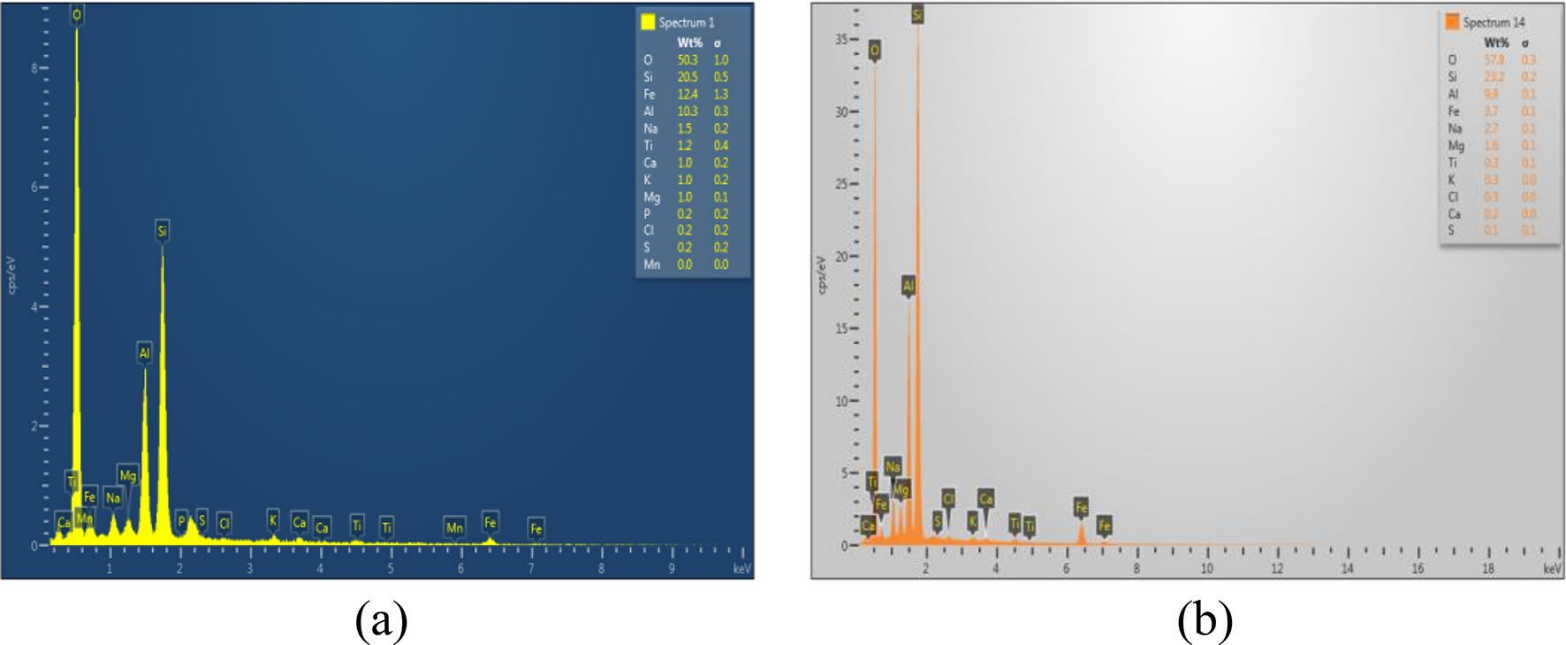
(**a**) EDX analysis of bentonite saturated with cement water (**b**) EDX analysis of bentonite saturated with distilled water.

Figure 11a,b show the EDX pattern of the bentonite saturated with hyperalkaline cement water and distilled water, respectively. The percentage of Na^+^ was observed to be reduced when saturated with hyperalkaline cement water, whereas the percentage of calcium showed a tremendous increase.

It is clear from the discussion in the preceding section that the specimens saturated with distilled water exhibited larger values of swell pressure and achieved full saturation with lesser time compared to the specimens saturated with the cement water. A probable reason for this observation was (1) due to the clogging of pores by the presence of secondary non- swelling minerals such as portlandite and (2) the precipitation of calcium in the specimen saturated with the hyperalkaline cement water. According to^53^ the presence of portlandite might have clogged the pores of the specimens saturated with the hyperalkaline cement water, leading to a reduction in the permeability. Hence, the time taken to reach all the accessible pores is more in these specimens saturated with cement water compared to the specimen saturated with the distilled water. The presence of portlandite is evident in Figs. 9 and 10a. More evidence on the reduction in permeability due to the calcium deposition that can be seen in Figs. 9 and 10b, where the lower portion of the specimen exhibited the presence of portlandite while the upper portion did not. Hence, the final swell pressure values were less in these specimens than the specimen saturated with the distilled water since monovalent cations in the porewater would impart more swelling than the divalent cations^57^.

## Conclusion

The eventual repository has the percolation of hyperalkaline cement water through the host rock reacting to the thermal history induced compacted bentonite layer. Hence the combined effect of the hyperalkaline environment and thermal history on the swell pressures of compacted bentonite was studied in this paper, which is not reported in the past. In this study, swelling pressure tests were conducted on compacted (1.5 Mg/m^3^, 1.75 Mg/m^3^, and 2.0 Mg/m^3^) Barmer bentonite specimens those were subjected to thermal history at 110 °C and 200 °C. The influence of thermal history and cement water on swelling behavior on the microstructure of compacted Barmer bentonites was analyzed and compared with the samples saturated with distilled water. The hyperalkaline (pH = 12.5) cement water reduced the swell pressure of both the compacted Barmer bentonites. The final swell pressure value of samples saturated with cement water was found to be approximately 30% less than that of samples saturated with distilled water. The induced thermal history was also responsible for the further reduction in final swell pressure values. The swell pressure decreased further as the temperature of the thermal history rose. The saturation time was nearly 1.5 times higher for cement water than for distilled water. The microstructural observations revealed that the clogging of the pores might have been occurred due to portlandite mineral and precipitation of calcite, thus increasing the time of saturation. The swell pressure values of bentonite B_2_ were less than that of B_1_ because of the difference in its initial characterization, especially liquid limit, cation exchange capacity, and specific surface area. The time of saturation taken in case of samples compacted at 2 Mg/m^3^ was more than that of samples compacted at lower densities for all thermal histories as well as for both the saturating solutions; therefore, it can be said that the higher density can delay the time of saturation of the compacted bentonite buffer in the deep geological repository and sustain more to the worst effect of hyperalkalinity and thermal history as well. This research, however, is laboratory tests that can be modeled to determine the long-term response of compacted bentonite to the thermal history and hyperalkalinity.

## Data Availability

All data, models, and code generated or used during the study appear in the submitted article.
